# Geospatial availability of breast cancer treatment modalities and hypothetical access improvement in Ghana: A nationwide survey

**DOI:** 10.1371/journal.pone.0291454

**Published:** 2023-09-15

**Authors:** Sarah Schoenhals, Meghan E. Mali, Edward K. Sutherland, Justin Sorenson, Florence Dedey, Jonathan Nellermoe, Angel Flores-Huidobro Martinez, Mamadou D. Tounkara, Raymond R. Price, Kirstyn E. Brownson

**Affiliations:** 1 Center for Global Surgery, University of Utah School of Medicine, Salt Lake City, Utah, United States of America; 2 Department of Surgery, University of Utah Health, Salt Lake City, Utah, United States of America; 3 Ensign Global College, Kpong, Eastern Region, Ghana; 4 J.Willard Marriot Library, University of Utah, Salt Lake City, Utah, United States of America; 5 Department of Surgery, University of Ghana Medical School, Accra, Greater Accra, Ghana; 6 Department of Surgery, Korle Bu Teaching Hospital, Accra, Greater Accra, Ghana; 7 General Surgery, Intermountain Medical Center, Murray, Utah, United States of America; 8 Department of Surgery, Huntsman Cancer Institute at University of Utah, Salt Lake City, Utah, United States of America; Sergio Arouca National School of Public Health: Escola Nacional de Saude Publica, BRAZIL

## Abstract

Breast cancer in Ghana is a growing public health problem with increasing incidence and poor outcomes. Lack of access to comprehensive treatment in Ghana may be a contributing factor to its high mortality. The purpose of this study was to evaluate the availability of treatments nationwide and systematically identify high yield areas for targeted expansion. We conducted a cross-sectional, nationwide hospital-based survey from November 2020-October 2021. Surveys were conducted in person with trained research assistants and described hospital availability of all breast cancer treatments and personnel. All individual treatment services were reported, and hospitals were further stratified into levels of multi-modal treatment modeled after the National Comprehensive Cancer Network (NCCN) Framework treatment recommendations for low-resource settings. Level 3 included Tamoxifen and surgery (mastectomy with axillary lymph node sampling); Level 2 included Level 3 plus radiation, aromatase inhibitors, lumpectomy, and sentinel lymph node biopsy; Level 1 included Level 2 plus Her2 therapy and breast reconstruction. Hospitals were identified that could expand to these service levels based on existing services, location and personnel. The distance of the total population from treatment services before and after hypothetical expansion was determined with a geospatial analysis. Of the 328 participating hospitals (95% response rate), 9 hospitals had Level 3 care, 0 had Level 2, and 2 had Level 1. Twelve hospitals could expand to Level 3, 1 could expand to Level 2, and 1 could expand to Level 1. With expansion, the population percentage within 75km of Level 1, 2 and 3 care would increase from 42% to 50%, 0 to 6% and 44% to 67%, respectively. Multi-modal breast cancer treatment is available in Ghana, but it is not accessible to most of the population. Leveraging the knowledge of current resources and population proximity provides an opportunity to identify high-yield areas for targeted expansion.

## Introduction

Breast cancer has become one of the most prevalent cancers and the most common cause of cancer-related death for women in Ghana [1, 2]. In 2020, there were an estimated 4,482 new cases (31.8% of all new cancer cases) in Ghana with over 2,000 breast cancer deaths [3]. Breast cancer is a growing public health challenge as incidence rises and outcomes show little improvement [2, 4]. Similar to other Sub-Saharan African countries [5], the 5-year survival for breast cancer in Ghana has been estimated at under 40% [6], whereas in high-income countries (HICs) such as the United States or Australia it is nearly 90% [4]. This striking disparity is attributed to numerous factors including socioeconomic, cultural, and geographic limitations combined with later stages at diagnosis and more aggressive subtypes [1, 6–9].

Breast cancer can be highly treatable with appropriate resources, as evidenced by more favorable outcomes in HICs [5]. Treatment of breast cancer requires a multi-disciplinary approach and varies based on stage, receptor status, treatment preference and patient factors such as age, menopausal status, and family history [10]. Surgery, a mainstay of management, includes resection either with mastectomy or lumpectomy and sampling of axillary lymph nodes. If lumpectomy is pursued, adjuvant whole breast radiation must be considered to reduce recurrence risk [10, 11]. Because of the high rate of estrogen and progesterone receptor-positive cancers worldwide, endocrine therapy such as Tamoxifen is an important treatment modality [12, 13]. Surgery with adjuvant endocrine therapy alone can be sufficient for certain early-stage cancers, but locally advanced or higher risk cancers may also require chemotherapy, biologic human epidermal growth factor receptor 2 (Her2)-targeted therapy, radiation to the chest wall or axillary nodes, and/or ovarian suppression [10, 14, 15]. Additionally, breast reconstruction is significantly associated with patient quality of life, and thus is considered an important aspect of breast cancer treatment [10, 16].

The National Comprehensive Cancer Network (NCCN) Framework™ is an evidence-based tiered system, consisting of Basic, Core, and Enhanced levels, which was developed to encompass screening, diagnosis, and treatment strategies for healthcare providers to identify appropriate options that could deliver the best possible outcomes in resource-constrained settings. Basic level of care is defined as the bare minimum care that will improve disease-specific outcomes, whereas the higher tiers include more resource-intensive modalities that can provide improvements in outcomes but may not be essential [12, 17]https://paperpile.com/c/5QwHYZ/FN3w9+S9V67. Basic treatment consists of mastectomy with axillary lymph node dissection and adjuvant

Tamoxifen, which are well established at improving survival in a significant percentage of breast cancers [10, 14, 17]. Thus, even in the absence of more advanced modalities, they may at least curtail more advanced or aggressive cancers. It has also been suggested that in the absence of hormone receptor testing, which is not widely available in low-resource settings, Tamoxifen should still be considered given its relatively low cost and low risk profile [12, 18].

The complexity of multidisciplinary treatment makes it challenging for patients to receive appropriate, comprehensive care in low- and middle-income countries (LMICs). Tamoxifen and mastectomies tend to be more widely available in LMICs, however resource-intensive modalities including radiation, chemotherapy, biologic treatment and surgical treatments such as axillary lymph node sampling or breast reconstruction are far more limited [14, 19, 20]. This pattern has been seen in Ghana, where two recent studies demonstrated that diagnosed patients were more likely to undergo surgical resection while completion of adjuvant treatments such as radiation, chemotherapy, and endocrine therapy was highly variable [21, 22]. Approximately 90% of breast cancers treated in Ghana have positive axillary lymph nodes [23], and the majority of patients present with late stage disease [6, 24, 25]. While receptor status testing is not always performed, when it has been done a higher incidence of triple negative breast cancers has been noted in Ghana [23, 26]. These factors strongly emphasize the need for access to treatment with locoregional control and multi-disciplinary adjuvant therapies.

Ghana is a middle-income country in sub-Saharan Africa with a population near 30 million people [27]. It is subdivided into 16 regions as of 2018, with most of the population residing in the Greater Accra and Ashanti regions [28–30]. The hospital system is tiered from tertiary referral or teaching hospitals to regional or district-level hospitals [28]. Even though there exists no formal specifications detailing what these different health facilities should provide in regards to breast cancer, comprehensive care is expected to be highest at tertiary teaching hospitals, followed by regional hospitals, municipal hospitals, then district hospitals [28, 31]. In 2011, the Ghanaian Ministry of Health (MOH) released a National Strategic Cancer Plan for 2012–2016 and set a goal to standardize treatment protocols and make all breast cancer treatment modalities available to all women in Ghana [32]. At that time, two hospitals were recognized as offering all treatment modalities [32]. The only existing national guideline for breast cancer care is in the MOH’s “Standard Treatment Guidelines” which covers a broad spectrum of diseases and a general aim to have all breast cancer treatment modalities available [33]. No further strategies or national guidelines have since been released. An updated assessment of available services is crucial in order to establish new priorities.

The purpose of this study was to determine the existing hospital-based treatment services and their geographic availability nationwide in Ghana, and to identify areas where targeted expansion of services would be most impactful.

## Materials and methods

### Study design

A cross-sectional, hospital-based survey designed to assess breast cancer care capacity was performed from November 16, 2020 to October 6, 2021. All health facilities in Ghana with a hospital designation were approached for participation in the study. Specialized hospitals that were not expected to provide breast cancer care, for example, orthopedic or psychiatric hospitals, were excluded. Lists of eligible hospitals were obtained and compiled from records of the Health Facilities Regulatory Agency and the Regional Health Directorates of the Ghana Health Services. Ethical Clearance was first sought from the Ghana Health Services Ethics Review Committee and additionally at the Institutional Review Boards of all academic institutions participating in the study.

### Survey design and structure

The survey aimed to comprehensively describe all hospital-based services and healthcare personnel available for the care of breast cancer in Ghana. The survey design was based on the World Health Organization’s Situational Analysis Tool to Assess Emergency and Essential Surgical Care and the Surgeons OverSeas Personnel, Infrastructure, Procedure, Equipment, and Supplies tool for assessing surgical infrastructure [34, 35]. The survey obtained information on the number of healthcare personnel employed at each hospital and the imaging, laboratory, screening, diagnostic, treatment, and follow-up services offered. NCCN Framework^TM^ resources were closely reviewed by our research team to ensure all screening, diagnostic, and treatment modalities relevant to care in limited resource settings were included [17, 36, 37]. A pilot study conducted in the Eastern Region of Ghana from March 2020 through May 2020 established the feasibility of an in-person hospital-based survey approach and identified survey questions that needed revision for clarity prior to nationwide roll-out [38]. The pilot survey was reviewed and revised by specialists in breast cancer care from both the United States and Ghana prior to initiation of the nationwide study. The expanded survey and guide detailing all sections of the survey is included as S1 Appendix. Of note, the survey included an assessment of cervical cancer care capacity, however, only breast cancer care is reported in this study.

### Training research assistants

Ten Ghanaian research assistants (RAs) were hired for the administration of the survey. All RAs were required to attend two training sessions led by researchers. Videos explaining each question of the survey and background information about breast and cervical cancer were available to the RAs throughout the study. All training was performed in a virtual format due to the COVID-19 pandemic.

### Survey administration and data collection

Letters describing the purpose of the survey were sent to all eligible hospitals from the office of the Director General of the Ghana Health Services through the Regional Health Directorates in November 2020. These letters explained that the study was supported by the GHS and the regional health directorates. After a copy of the survey and a letter were sent, the RAs called each hospital to arrange a time to meet with a survey respondent in person at the hospital to obtain data via a structured interview. Informed written consent was obtained for each hospital by the designated respondent. Respondents selected at each hospital included key administrative personnel, the most knowledgeable clinical specialist of the facility, or the lead breast cancer specialist. If a question was encountered that the respondent did not know, the appropriate person within the hospital was contacted, and RAs would arrange a time to follow up with each hospital to acquire necessary missing information. Data was collected on paper by the RAs, and then later entered in an electronic form that was accessible only to members of the research team. All data entries were reviewed. Missing data and inconsistent responses were reviewed with the corresponding RAs who then clarified the data entry or re-contacted the hospitals for clarification. All hospitals involved in the initial pilot study were re-surveyed.

### Defining terms

The primary focus of this paper was to outline individual treatment modalities at each hospital and to identify which hospitals offered multi-disciplinary approaches. Treatment modalities included surgery, radiation, chemotherapy, biologic Her2-directed therapy and endocrine therapy. “Basic surgery” was defined as a minimum of mastectomy with axillary lymph node sampling either via level I/II axillary dissection or sentinel node biopsy. Hospitals that offered mastectomy without axillary management are reported but were not classified as offering basic surgery. Lumpectomy by NCCN guidelines should be followed with adjuvant whole breast radiation, therefore lumpectomy alone was not considered a component of basic surgery [10]. Chemotherapy was subdivided into individual agents and combination therapy, which could include per NCCN Framework™ various regimens of Cyclophosphamide, Methotrexate, 5-Fluorouracil, Anthracyclines and/or Taxanes [10, 37]. Her2 targeted therapy included Trastuzumab and Lapatinib. Endocrine therapy was subdivided into individual endocrine agents and ovarian suppression via surgical oophorectomy, medication, or irradiation. Radiation therapy included breast, chest wall, and nodal radiation.

### Hospital stratification

Hospitals that offered treatments were stratified using the NCCN Framework™ as a model. This was used to demonstrate which hospitals offered multi-disciplinary treatment using an international standard, in the absence of national guidelines. While the framework includes screening, diagnostic, and treatment components, only treatment options were examined in this paper. Levels 3, 2, and 1 were equivalent to the NCCN Basic, Core and Enhanced tiers for breast cancer treatment, respectively (Table 1). Hospitals must offer all minimum required services to be considered at that level of care. Hospitals were identified as hypothetically capable of providing Levels 3, 2, or 1 of service if they had appropriate staff on site and needed at most 1–2 additional services to meet the higher level of care. Appropriate staff included at least one general surgeon and anesthesia provider, and, if expansion would include breast reconstruction, a plastic surgeon must also have been on staff. For any expansion of surgical services, the hospital must already have offered some form of surgical treatment (such as mastectomy without axillary node dissection), thus already theoretically having appropriate resources to provide surgical care. These hospitals were also required to be either in a region without that level of service, or greater than 45 kilometers from any other hospitals with that level of service. All hospitals were de-identified for reporting.

**Table 1:** Hospital stratification system.

Designation	NCCN Framework™ Equivalent	Minimum Treatment Services Required
**Level 3**	Basic	◾ Level I/II Axillary Dissection ◾ Total Mastectomy ◾ Tamoxifen
**Level 2**	Core	*Level 3/Basic services*, *plus*: ◾ Breast Conserving Surgery (Lumpectomy) ◾ Sentinel Lymph Node Biopsy ◾ Aromatase Inhibitors ◾ Chemotherapy ◾ Radiation Therapy
**Level 1**	Enhanced	*Level 2/Core & Level 3/Basic services*, *plus*: ◾ Breast reconstruction ◾ Her2 directed therapy

Lumpectomy should be followed with adjuvant radiation

Aromatase Inhibitors = Anastrozole, Letrozole, Fulvestrant, Exemestane

Chemotherapy = Combinations of either Cyclophosphamide, Methotrexate, 5-Fluorouracil (CMF); 5-Fluorouracil, Doxorubicin, Cyclophosphamide (FAC); Doxorubicin, Cyclophosphamide (AC); OR Epirubicin, Cyclophosphamide (EC); with OR without additional therapies including Taxanes (Docetaxel or Paclitaxel).

### Data analysis

The survey results are presented overall as frequencies and percentages. Analysis was performed using Stata version 17.0 (StataCorp, revised 2022). ArcGIS Pro Software (Environmental Systems Research Institute v2.9.2, 2021) was used to create maps with proximity buffers extending outward in 5 kilometer (km) increments. For population analysis, the landmass and population of each health region was estimated using the 2020 LandScan population density data set from Oak Ridge National Laboratory (Oak Ridge, TN, USA). The entire population of Ghana and each region were used. A zonal statistics tool was then used to estimate the percentage of the population that lived within specific Euclidean distances of hospitals. Because there was no data available to account for specific travel times, road conditions, or other geographic barriers in Ghana, Euclidean distances were used at increments of 25km, 45km and up to 75km. These were aimed to provide an estimate of travel times from less than 30 minutes up to greater than 2 hours, with the understanding that several other factors and barriers can exist to elongate travel times within these distances. Individual treatment modalities (surgery, hormone suppression, chemotherapy and radiation) as well as treatment Levels 1, 2, 3 and hypothetical levels of treatment were included in the mapping and population analysis.

## Results

Three hundred and forty-six hospitals across Ghana were identified for possible participation in the survey, of which 328 were surveyed with an overall response rate of 94.8%. Of the 18 hospitals that were not surveyed, 15 declined to participate, 2 were closed for renovation, and 1 could not be located. All 16 regions had hospitals included in the survey, ranging from 88 (26.8%) in the Greater Accra region to 3 (0.9%) in the Savannah region. The hospital types consisted of 88 (26.8%) district hospitals, 39 (11.9%) municipal hospitals, 8 (2.4%) regional hospitals, 7 (2.1%) teaching hospitals, 3 (0.9%) metropolitan hospitals, and 183 (55.8%) hospitals without specific additional designation. Of the 328 hospitals that completed the survey, 321 (97.9%) reported seeing breast cancer patients and 40 (12.2%) hospitals offered at least one breast cancer treatment service. Most hospitals offering treatment were in Greater Accra (16, 40.0%) and Ashanti (6, 15.0%) regions. Breast cancer treatments were not available in the Ahafo, Bono East, Oti, Savannah, and Western North regions (Table 2).

**Table 2:** Summary of regions and hospitals in survey. Population and Regional Area Based on 2020 Landscan Data.

Region	Area in km^2^	Population of region n, (% of total population)	Hospitals surveyed n (% of total hospitals surveyed)	Hospitals with breast cancer treatment services n (% of hospitals with treatment services)
**Ahafo (AHR)**	5,193	580,916 (2.0%)	4 (1.2%)	0
**Ashanti (ASR)**	24,389	5,608,914 (19.1%)	70 (21.3%)	6 (15.0%)
**Bono (BR)**	11,107	1,121,771 (3.8%)	12 (3.7%)	3 (7.5%)
**Bono East (BER)**	23,257	1,073,680 (3.7%)	11 (3.4%)	0
**Central (CR)**	9,826	2,479,716 (8.5%)	21 (6.4%)	3 (7.5%)
**Eastern (ER)**	19,323	3,142,749 (10.7%)	34 (10.4%)	2 (5.0%)
**Greater Accra (GAR)**	3,245	4,769,448 (16.3%)	88 (26.8%)	16 (40.0%)
**North East (NER)**	9,074	558,199 (1.9%)	3 (0.9%)	1 (2.5%)
**Northern (NR)**	25,448	1,845,972 (6.3%)	13 (4.0%)	1 (2.5%)
**Oti (OR)**	11,066	720,175 (2.5%)	6 (1.8%)	0
**Savannah (SR)**	35,862	562,962 (1.9%)	3 (0.9%)	0
**Upper East (UER)**	8,842	1,240,459 (4.2%)	11 (3.4%)	2 (5.%)
**Upper West (UWR)**	18,476	837,940 (2.9%)	10 (3.0%)	1 (2.0%)
**Volta (VR)**	9,504	1,804,547 (6.2%)	16 (4.9%)	3 (7.5%)
**Western (WR)**	13,847	2,079,737 (7.1%)	18 (5.5%)	2 (5.0%)
**Western North (WNR)**	10,074	899,320 (3.1%)	8 (2.4%)	0
**16 regions**	**238,533 km** ^**2**^	**Total population: 29,326,505**	**328 hospitals surveyed **	**40 hospitals with breast cancer treatment services **

### Surgery

Basic surgery was available at 33 hospitals across 9 regions, and was not available in Ahafo, Bono East, Eastern, North East, Oti, Savannah, or Western North regions. Ten of those 33 hospitals provided sentinel lymph node biopsy. Thirty-two of those 33 hospitals offered lumpectomy as well, of which 2 offered adjuvant whole breast radiation therapy. Nine of all surveyed hospitals provided options for breast reconstruction. Three hospitals offered mastectomy, lumpectomy, axillary dissection, sentinel node biopsy, and breast reconstruction. Two of these three hospitals were located in Greater Accra and one was located in the Ashanti region. Forty-five additional hospitals offered total mastectomy and/ or lumpectomy, but they were not classified as offering “basic surgery” due to their lack of axillary management (Table 3).

**Table 3:** Number and frequency of hospitals and regions with each different treatment service.

Service Offered	Hospitals with service, n = 328 (%)	Regions with service, n = 16 (%)
**Surgery** ◽ Mastectomy ◽ Lumpectomy ◽ Axillary dissection ◽ Sentinel lymph node biopsy ◽ Breast reconstruction	50 (15.2%) 78 (23.8%) 33 (10.1%) 10 (3.0%) 9 (2.7%)	12 (75.0%) 13 (81.3%) 9 (56.3%) 4 (25.0%) 4 (25.0%)
**Hormone Therapy** ◽ Tamoxifen ◽ Anastrozole ◽ Letrozole ◽ Exemestane ◽ Fulvestrant ◽ Fluoxymesterone ◽ Oophorectomy ◽ Ovarian irradiation ◽ Medical functional ovarian suppression (Leuprolide/Goserelin)	15 (4.6%) 13 (4.0%) 6 (1.8%) 4 (1.2%) 4 (1.2%) 1 (0.3%) 9 (2.7%) 3 (0.9%) 9 (2.7%)	8 (50.0%) 7 (43.8%) 4 (25.0%) 3 (18.8%) 3 (18.8%) 1 (6.3%) 6 (37.5%) 2 (12.5%) 4 (25.0%)
**Chemotherapy** ◽ Combination either CMF, FAC, AC, EC +/- Taxanes ◽ Cyclophosphamide ◽ 5-Fluorouracil ◽ Methotrexate ◽ Anthracyclines (Doxorubicin or Epirubicin) ◽ Taxanes (Paclitaxel or Docetaxel) ◽ Capecitabine ◽ Gemcitabine ◽ Platinum-based agents (Cisplatin or Carboplatin) ◽ Vinorelbine	18 (5.5%) 18 (5.5% 16 (4.9%) 8 (2.4%) 15 (4.6%) 11 (3.4%) 10 (3.0%) 3 (0.9%) 4 (1.2%) 2 (0.6%	8 (50.0%) 8 (50.0%) 8 (50.0%) 5 (31.3%) 7 (43.8%) 6 (37.5%) 6 (37.5%) 2 (12.5%) 2 (12.5%) 1 (6.3%)
**Biologic Her2 targeted therapy** ◽ Trastuzumab ◽ Lapatinib	6 (1.8%) 2 (0.6%)	4 (25.0%) 2 (12.5%)
**Radiation** (Whole Breast, Chest Wall, Nodal)	3 (0.9%)	2 (12.5%)

### Systemic therapy

Sixteen hospitals offered endocrine therapy. One offered aromatase inhibitors only, while 15 had Tamoxifen with or without other agents. Tamoxifen was available across the Ashanti, Bono, Central, Eastern, Greater Accra, Northern, Volta, and Western regions. Aromatase inhibitors were available in 14 hospitals across the Ashanti, Bono, Central, Eastern, Greater Accra, Volta, and Western regions. Twelve hospitals offered some form of ovarian suppression, 6 of which also offered endocrine therapy. Eighteen hospitals offered one or more standard forms of combination chemotherapy. Chemotherapy was not offered in Ahafo, Bono, Bono East, Oti, Savannah, Upper East, Upper West, or Upper North regions. Her-2 targeted therapy was available at 6 hospitals in the Ashanti, Central, Greater Accra, and Volta regions (Table 3).

### Radiation

Three hospitals, two in Greater Accra and one in Ashanti, offered radiation therapy with whole breast, chest wall, and nodal radiation (Table 3).

### Healthcare personnel

Every region except Savannah had at least one general surgeon, with the highest concentration of general surgeons in Greater Accra (63 hospitals) followed by Ashanti (34 hospitals). Anesthesia providers, either medical doctors (MD) or certified nurse anesthetists (CNA), were in every region employed across 227 (69.2%) hospitals. Sixteen hospitals reported offering surgery, but did not have a surgeon on staff, while 12 hospitals reported offering surgery without anesthesia on staff. Further investigation indicated staff was called in on an as-needed basis from other hospitals. Eight hospitals had an oncologist MD on staff, and all but 14 of the hospitals surveyed reported a general practitioner physician (Table 4). Of importance, the scope of practice of general surgeons, oncologists and general practitioners in terms of medical or surgical management of breast cancer was not assessed, so this survey is not indicative of what role they may have in breast cancer treatment. Anecdotally, it was described that surgeons often manage medical treatments for breast cancer as did general practitioners in various facilities.

**Table 4:** Summary of hospital personnel potentially involved in breast cancer treatment nationwide.

Provider type	Total number of providers	Number of hospitals with at least one provider *n = 328 (%)*	Regions with at least one provider, *n = 16 (%)*
**General Surgeon**	366	179 (54.6%)	15 (93.8%)
**Anesthesia (MD or CRNA)**	726	227 (69.2%)	13 (81.3%)
**Oncologist**	14	8 (2.5%)	4 (25.0%)
**Plastic Surgeon**	57	28 (8.4%)	7 (43.8%)
**General Practitioner**	2,431	314 (95.7%)	16 (100%)

### Multimodal care & hypothetical expansion

There were 9 hospitals in the Central, Greater Accra, Northern, Volta and Western regions that offered Level 3 treatment modalities (Table 5). A variety of other services were available in some of these Level 3 hospitals: 7 hospitals offered chemotherapy, 7 offered treatment with aromatase inhibitors, 3 administer Her2 directed therapy, 3 perform sentinel node biopsy, and 3 perform breast reconstruction. No hospitals offered Level 2 care. Level 1 care was available in two hospitals in the Greater Accra and Ashanti regions. All hospitals had at least 1 general surgeon, anesthesia provider and general practitioner on site. Of note, neither of the level 1 hospitals had a medical oncologist reported on staff, however on further questioning at each facility it was presumed that medical oncologic practice was under the scope of the other providers listed within the facility.

**Table 5:** Individual hospitals with Level 3, 2, and 1 care, personnel and additional services offered. Hospitals are Randomly Assigned a Letter.

Treatment Level	Hospital	Region	Additional treatment services offered beyond requirement for Level	Hospital Personnel on staff at facility
Level 3* (NCCN Basic)	A	Central	Chemotherapy Aromatase Inhibitors Sentinel Lymph Node Biopsy Her2 Therapy	12 General Surgeons 1 Plastic Surgeon 2 Anesthesia Providers 36 General Practitioners
	B	Greater Accra	Chemotherapy Aromatase Inhibitors	1 General Surgeon 1 Plastic Surgeon 2 Anesthesia Providers 1 Oncologist 6 General Practitioners
	C	Greater Accra	Aromatase Inhibitors Breast Reconstruction	3 General Surgeons 1 Plastic Surgeon 3 Anesthesia Providers 20 General Practitioners
	D	Greater Accra	Chemotherapy Aromatase Inhibitors Breast Reconstruction	2 General Surgeons 1 Plastic Surgeon 7 Anesthesia Providers 65 General Practitioners
	E	Greater Accra	Chemotherapy Aromatase Inhibitors Her2 Therapy	8 General Surgeons 2 Plastic Surgeons 28 Anesthesia Providers 80 General Practitioners
	F	Northern	Chemotherapy	7 General Surgeons 1 Plastic Surgeon 45 Anesthesia Providers 140 General Practitioners
	G	Western	Aromatase Inhibitors Breast Reconstruction	1 General Surgeon 9 Anesthesia Providers 1 Oncologist 6 General Practitioners
	H	Volta	Chemotherapy	2 General Surgeons 4 Anesthesia Providers 10 General Practitioners
	I	Volta	Chemotherapy Aromatase Inhibitors Her2 Therapy	6 General Surgeons 12 Anesthesia Providers 4 Oncologists 10 General Practitioners
**Level 2 (NCCN Core)**	None	n/a	n/a	
**Level 1 (NCCN Enhanced)**	J	Ashanti		22 General Surgeons 7 Plastic Surgeons 13 Anesthesia Providers 223 General Practitioners
	K	Greater Accra		25 General Surgeons 18 Plastic Surgeons 19 Anesthesia Providers 207 General Practitioners

Each level 3 hospital offered simple mastectomy, axillary lymph node dissection, and endocrine therapy with tamoxifen. Level 2 hospitals offer Level 3 service plus lumpectomy, sentinel lymph node biopsy, aromatase inhibitors, combination chemotherapy, radiation therapy. Level 1 hospitals offer all Level 1 and Level 2 services in addition to Her2 therapy and breast reconstruction.

**Fig 1:**
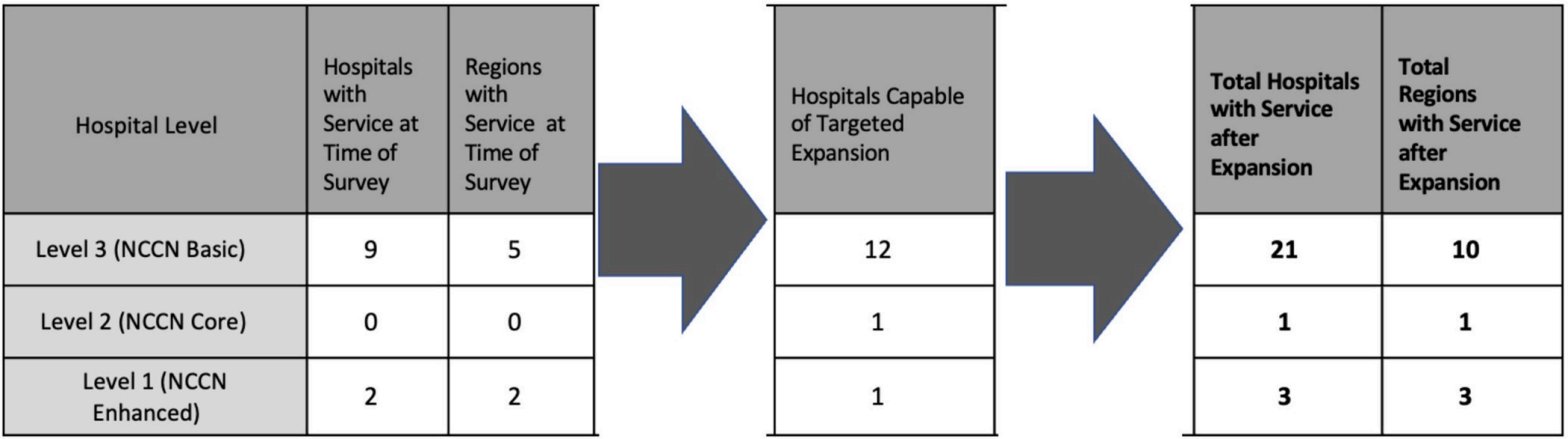
Hospitals and Regions with Multi-disciplinary Care at Time of Survey and with Hypothetical Expansion. (A) Locations of hospitals with Levels 1, 2, 3 treatment at time of survey. (B) Locations of hospitals with Levels 1,2, 3 treatment with hypothetical expansion. Basemap: https://www.arcgis.com/home/item.html?id=b9b1b422198944fbbd5250b3241691b6. National Geographic, Esri, DeLorme, HERE, UNEP-WCMC, USGS, NASA, ESA, METI, NRCAN, GEBCO, NOAA, iPC [39].

**Fig 2:**
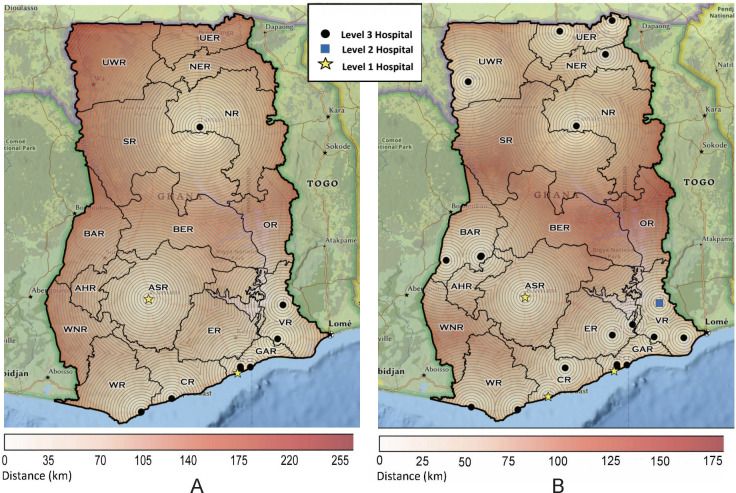
Ghanaian hospitals with Levels 1–3 of multidisciplinary treatment at time of survey, and with hypothetical expansion.

With targeted expansion of one or two services, 12 hospitals in 5 additional regions including Bono, Eastern, North East, Upper East and Upper West regions were hypothetically capable of advancing to Level 3 status (Fig 1). Eight of these required the addition of Tamoxifen only to advance while 2 required axillary dissection only and 2 required both axillary dissection and Tamoxifen (Table 6). All hospitals requiring expansion of surgical capacity had general surgeons and anesthesia providers employed on site. This hypothetical expansion increased the total number of hospitals capable of providing Level 3 breast cancer treatment from 9 to 21 (Figs 1, 2). There were 2 Level 3 hospitals found to be capable of expanding to a higher level of treatment. Hospital I in the Volta region could advance to a Level 2 status with the addition of radiation and sentinel node biopsy. Hospital A in the Central region could expand to Level 1 status with addition of radiation and breast reconstruction. This hospital reported a plastic surgeon employed on site at the time of the survey.

**Table 6:** Individual hospitals with potential for targeted expansion based on existing services, location and personnel. Hospitals are Assigned a Random Letter.

Target Level	Hospital Letter	Region	Existing Hospital Services	Services Needed for Expansion to Target Level	Existing Hospital Personnel
**Level 3 (NCCN Basic):**	L	Bono	Basic Surgery	Tamoxifen	1 General Surgeon 8 Anesthesia Providers 16 General Practitioners
	M	Bono	Basic Surgery	Tamoxifen	1 General Surgeon 3 Anesthesia Providers 6 General Practitioners
	N	Bono	Mastectomy Tamoxifen Aromatase Inhibitors	Axillary Lymph Node Dissection	1 General Surgeon 4 Anesthesia Providers 12 General Practitioners
	O	Eastern	Mastectomy Tamoxifen Chemotherapy Aromatase Inhibitors Breast Reconstruction	Axillary Lymph Node Dissection	2 General Surgeons 3 Anesthesia Providers 5 General Practitioners
	P	Eastern	Mastectomy Chemotherapy	Axillary Lymph Node Dissection Tamoxifen	3 General Surgeons 6 Anesthesia Providers 48 General Practitioners
	Q	Central	Basic Surgery	Tamoxifen	1 General Surgeon 5 Anesthesia Providers 4 General Practitioners
	R	North East	Mastectomy Chemotherapy	Axillary Lymph Node Dissection Tamoxifen	1 General Surgeon 2 Anesthesia Providers 2 General Practitioners
	S	Upper East	Basic Surgery	Tamoxifen	2 General Surgeons 12 General Practitioners
	T	Upper East	Basic Surgery	Tamoxifen	12 General Practitioners
	U	Upper West	Basic Surgery	Tamoxifen	3 General Surgeons 3 Anesthesia Providers 5 General Practitioners
	V	Volta	Basic Surgery Chemotherapy	Tamoxifen	1 General Surgeon 3 Anesthesia Providers 7 General Practitioners
	W	Western	Basic Surgery Sentinel Node Biopsy Chemotherapy	Tamoxifen	1 General Surgeon 2 Anesthesia Providers 2 Oncologists 4 General Practitioners
**Level 2 (NCCN Core)**	I	Volta	Basic Surgery Tamoxifen Chemotherapy Aromatase Inhibitors Her 2 Therapy	Sentinel lymph node biopsy Radiation Therapy	6 General Surgeons 12 Anesthesia Providers 4 Oncologists 72 General Practitioners
**Level 1 (NCCN Enhanced)**	A	Central	Basic Surgery Tamoxifen Chemotherapy Aromatase Inhibitors Sentinel Lymph Node Biopsy Her2 Therapy	Breast Reconstruction Radiation Therapy	12 General Surgeons 1 Plastic Surgeon 2 Anesthesia Providers 36 General Practitioners

Basic Surgery = Total Mastectomy *AND* Axillary Lymph Node Dissection

Chemotherapy = Combination of either CMF, FAC, AC, EC with or without additional therapy including Taxanes (Docetaxel or Paclitaxel).

### Population analysis

Population analysis for individual treatment modalities demonstrated that less than 50% of the population were living within 25 kilometers (km) of each individual service. Conversely, 83.3% of the total population was within 75km of basic surgery, 69.1% within 75km of endocrine therapy, 71.5% within 75km of chemotherapy, and 43.8% were within 75km of radiation treatment (Table 7). For levels of care, 43.6% of the population lived within 75 km of Level 3 treatment services, while 41.8% lived within 75 km of Level 1 services (Table 8). With targeted expansion of services and the addition of 12 hospitals to the level 3 cohort, 65.9% of the population would be within 75km (increase by 22.4%), and 35.9% of the population would be within 25 km (11.7% increase). With expansion of one hospital to meet level 2 care, up to 1.2% and 6.1% of the population would be within 25 km and 75 km of these services, respectively. Expanding services for 1 hospital to meet level 1 care hypothetically would increase access within 25 km and 75 km by 1.7% and 7.7%, respectively (Table 8).

**Table 7:** Population within varied distances to each treatment service.

Service	Number of Hospitals with Service	Regions with Service	Population living within specified Euclidean distance n (% total population) total population 2020 = 29,326,505				
			0–25 km	26–45 km	46–75 km	Total within 75km	>75 km
**Basic surgery**	33	8	13,478,237 (46.0%)	5,757,299 (19.6%)	5,193,587 (17.7%)	**24,429,123 (83.3%)**	4,897,382 (16.7%)
**Endocrine therapy**	16	9	11,364,987 (38.8%)	3,513,036 (12.0%)	5,387,465 (18.4%)	**20,265,488 (69.1%)**	9,061,017 (30.8%)
**Chemotherapy**	18	8	11,582,923(39.5%)	3,482,358 (11.9%)	5,901,780 (20.1%)	**20,967,061 (71.5%)**	8,359,444 (28.5%)
**Radiation therapy**	3	2	8,052,413 (27.5%)	1,653,549 (5.6%)	3,145,803 (10.7%)	**12,851,765 (43.8%)**	16,474,740(56.1%)

**Table 8:** Population within varied distance to each level of care, and hypothetical population within each distance if targeted expansion is achieved.

Level of Service	Number of Hospitals with Level of Service	Regions with Level of Service	Population living within specified Euclidean distance n (% total population) total population 2020 = 29,326,505				
			0–25 km	25–45 km	46–75 km	Total within 75km	>75 km
**Level 3 (NCCN Basic)**	9	5	7,100,043 (24.2%)	2,290,279 (7.8%)	3,393,000 (11.6%)	12,783,32 (43.6%)	16,543,183 (56.4%)
**Total Hypothetical Level 3 with expansion**	21	10	10,534,080 (35.9%)	4,944,744 (16.9%)	3,851,808 (13.1%)	19,330,63 (65.9%)	9,995,873 (34.0%)
**Difference**	**+ 12**	**+5**	**+3,434,037 (11.7%)**	**+2,654,46 (9.1%)**	**+ 458,808 (1.5%)**	**+6,547,31 (22.4%)**	
**Level 2 (NCCN Core)**	0	0	**-**	**-**	**-**	**-**	**-**
**Total Hypothetical Level 2 with expansion (Difference)**	**+1**	**+1**	**+ 339,045 (1.2%)**	**+331,621 (1.1%)**	**+1,116,826 (3.8%)**	**+1,787,48 (6.1%)**	**+27,539,022 (93.9%)**
**Level 1 (NCCN Enhanced)**	2	**2**	7,271,292 (24.8%)	2,012,822 (6.9%)	2,966,380 (10.1%)	12,250,49 (41.8%)	17,076,011 (58.2%)
**Total Hypothetical Level 1 with expansion**	3	3	7,778,331 (26.5%)	2,420,051 (8.3%)	4,324,609 (14.7%)	14,522,991 (49.5%)	14,803,514 (50.5%)
**Difference**	**+1**	**+1**	**+507,039 (1.7%)**	**+ 407,229 (1.4%)**	**+1,358,22 (4.6%)**	**+2,272,49 (7.7%)**	

Table displays number of hospitals with each level designation and total number of hospitals that could have the level designation after targeted expansion described below. Displayed as total number of people and percentage of total population within specified Euclidean distance. Differences displayed as either increase(+) or decrease (-) of each category

## Discussion

Treatment of breast cancer and long-term survival is most successful with comprehensive, multi-disciplinary care that is tailored to individual patients. To our knowledge, this is the first study within Ghana to establish a baseline of existing treatment availability and set the stage for targeted improvements. We found that comprehensive treatment is offered in Ghana, but the vast majority of the population must travel long distances to access it. More so, the availability of each treatment modality is highly variable throughout the country. As it is now, a person diagnosed with breast cancer in Ghana, particularly in rural Ghana, may have to travel several hours and to multiple different locations to receive optimal locoregional and systemic treatment of their disease.

Just two urban hospitals offer Level 1 or NCCN Framework™ Enhanced level treatment at the time of our survey, which is unchanged from the Ghana National Cancer Strategy Release in 2011 [32]. Less than half the population is within 75 kilometers (km) of these two hospitals. Level 3 (NCCN Framework™ Basic) treatment includes Tamoxifen and basic surgery (mastectomy and axillary lymph node dissection), which are the mainstays of treatment to improve breast cancer survival, yet similarly, less than half the Ghanaian population is within 75km of a hospital with both of those services available. While we cannot estimate exact travel times based on Euclidean distance, we can estimate that 75 km is likely greater than 2 hours of travel time and 45km is greater than 1 hour. The Lancet Commission on Global Surgery recommends that a minimum of 80% of the population should be within 2 hours of essential surgery [40] and another study found that 80% of women in Ghana were unlikely to travel for medical care if treatment required greater than 1 hour of travel time [41]. Thus, most breast cancer patients, particularly those in rural areas or with limited travel means, are at high risk for fragmented or incomplete care which makes them vulnerable to suboptimal outcomes.

The survey also highlighted that there are several hospitals offering incomplete locoregional control. Many hospitals reported offering mastectomy or lumpectomy, but no axillary node sampling. Additionally, lumpectomy alone is widely offered, but not adjuvant radiation. The absence of these treatments may result in the undertreatment of many women. This emphasizes the importance of national guidelines for treatment standards and targeted expansion of services to encompass those standards.

### Potential for growth

While guidelines and targeted programs have shown success in HICs, the resource intensity required to improve breast cancer treatment makes it very difficult to make advancements in LMICs, and there is a paucity of well-described successful programs [42]. The World Health Organization (WHO) Breast Health Global Initiative (BHGI) has pioneered approaching this burden in LMICs through defining priorities based on resource availability [12, 18]. With stratified guidelines tailored to geographical resource settings and cancer stages, success becomes far more feasible [18]. The NCCN Framework™ was developed on this similar theme to emphasize the most essential care with a stepwise improvement approach [17]. With this study, we implemented the framework both in assessing existing treatment infrastructure and in assessing its utility for meaningful change.

The results of this study, specifically its determination of resources and personnel available nationwide, can be leveraged to create targeted and cost-effective strategies to expand access to a higher level of care for breast cancer patients in Ghana. This is essential to improve breast cancer survival in Ghana. Our survey found that only 33 hospitals offered basic surgery (mastectomy and axillary lymph node sampling), however, 179 hospitals reported at least one general surgeon on staff and even more had anesthesia providers. This alone could be a point of focus for expanding general surgical capabilities for breast cancer treatment nationwide. Of importance, general surgeons in Ghana who perform breast cancer surgery often also manage medical aspects of breast cancer treatment such as chemotherapy or endocrine therapy. This could mean with additional training or resource allocation, these services could expand further as well. As there are far fewer hospitals with oncologists and the role of general practitioners in breast cancer management is not well-established, it is more difficult to ascertain capabilities based on our understanding of these personnel with this study.

To identify the potentially highest yield for targeted expansion, our study focused on hospitals with the potential to offer multi-disciplinary Level 1, 2, or 3 care based on their existing services, location, and personnel. With specific interventions, there is potential for a large increase in the population to gain access to Level 3 care. Surgery is resource intensive, however, we found that several hospitals with general surgeons on staff offered mastectomy or lumpectomy but not axillary lymph node dissection. With additional surgical training, surgical care meeting the level of NCCN Framework^TM^ Basic guidelines could be offered at those hospitals. Additionally, Tamoxifen is a relatively low-cost medication that does not require frequent monitoring, has significantly few side effects compared to chemotherapy, and is well established to reduce the risk of recurrence and improve survival of hormone receptor-positive cancers [13, 19]. With a focus on adding Tamoxifen to 10 hospitals and increased surgical training to add axillary dissection to 4 hospitals, 53% of the Ghanaian population could be within 1 hour (45km) of a single hospital with Basic level of breast cancer treatment as compared to the 32% of the population currently.

Bearing in mind that there is a known high rate of triple-negative tumors as well as a high rate of late-stage cancers in Ghana [5, 24, 25], we must acknowledge that while targeting Level 3 tiered care is an important first step in tackling breast cancer treatment, this will not offer adequate treatment for high risk or advanced breast cancers. Thus, while expanding Level 3 care for a larger percentage of the population is an important first step, more must be done to improve survival rates for many breast cancer patients in Ghana. Access to higher-level, more comprehensive care proves to be more complicated. With targeted expansion to the three hospitals identified as capable of reaching Level 1 or Level 2 care, the increase in population within reasonable access was minimal. Given the intensity of resources (such as radiation) needed to expand to these levels this may not be a high-yield area to target for improvement. Other interventions may be more cost-effective to improve access to this level of care, such as transportation infrastructure or programs to support long-distance travel for patients and their families. Alternatively, identifying certain areas based on population density, rather than geographic location as was done in this study, may expand access to a larger portion of patients. Beyond that, strategies to improve early detection and treatment-seeking may also be more cost-effective means to improving outcomes than expanding access to Level 1 and Level 2 care. If increased numbers of patients present with early-stage cancers, fewer patients will require higher levels of service to treat their disease.

The purpose of this study is to set the tone for addressing the hypothetical expansion of breast cancer resources and assessing the underlying barriers to the lack of required structural capacity of targeted hospitals. It is equally important to note that there are several other socioeconomic, political, and cultural factors that should be explored in future studies- from an individual patient to public health viewpoint. Understanding the financial aspects, cultural norms, and social circumstances that affect a patient’s ability to get care is essential to improving outcomes and care access.

### Limitations

We had limitations with respect to our survey tool, hospital stratification system, criteria for expansion, and ability to do a targeted population analysis. While our survey tool was piloted in a previous study, it is not a previously validated tool, as no validated survey tool exists to our knowledge. Additionally, the reported hospital personnel at each hospital could be influenced by reporting bias, as unit personnel could be engaging in locum practices at multiple hospitals or be engaged as permanent staff in multiple hospitals. Similarly, because there are not defined titles for which providers manage the non-surgical aspects of breast cancer care, the data reported may not give an adequate assessment of the manpower available to provide breast cancer treatments described. Because of this, personnel were less heavily utilized in our identification of hospitals capable of expansion (only the presence of surgeons or anesthesia staff). Our criteria for expansion was additionally limited by not directly evaluating each hospitals’ existing equipment and physical infrastructure. This we ameliorated by using their existing services offered (such as surgery or chemotherapy) as a representative of infrastructure and resources making them more likely to be capable of expansion.

The adapted use of tiered NCCN Framework™ was limited in that we only included curative treatment options as part of the stratification system. We intentionally adapted the framework for treatment services to allow wide-spread inclusion of hospitals, which means certain hospitals were included despite possibly missing vital services such as palliative care or screening and diagnostics. It is recognized that important personnel and services in breast cancer treatment including pathology and staging were not included in our study, however these are the focus of a complementary study that is currently in progress.

Last, because of the lack of established data on demographics and breast cancer incidence in the individual regions of Ghana, our population included the entire population of each region of the nation, rather than a targeted population. This allowed us to account for all possible patients susceptible to breast cancer (including young people and males), but it made our population analysis less specific to breast cancer patients only.

## Conclusion

Access to appropriate multi-disciplinary breast cancer treatment is limited throughout Ghana, with striking gaps in geographic proximity to even the most basic cares. By leveraging our understanding of what is currently available to the population, we identified that there are potentially cost-effective means to expand access to basic treatments for most of the Ghanaian population. Improving access to higher-resource care is far more difficult. This study highlights the importance of a strategic, multi-faceted approach to expand access to care with the use of stratified guidelines, with the aim of improving breast cancer outcomes by minimizing delays in the patient care continuum.

## Supporting information

S1 Appendix Survey and question guide. (PDF)
