# Supplementary material for: Geospatial availability of breast cancer treatment modalities and hypothetical access improvement in Ghana: A nationwide survey
Source: PLoS One. 2023 Sep 15;18(9):e0291454. doi: 10.1371/journal.pone.0291454 (PMC10503733; doi:10.1371/journal.pone.0291454)
Supplement: S1 Appendix — (PDF) [file pone.0291454.s001.pdf]

S1 Appendix: Survey and Question Guide

Breast and Cervical PIPES Data Entry **Date ________ RA Initials______**

Section A – 1: Basic Information

1. Region:
2. District:
3. Date:
4. Respondent Name:
5. Respondent Phone:
6. Title/Position:
7. Facility:
8. Address:
9. GPS Lat: ___________Long: ____________
10. Facility Phone:
11. Facility Email:
12. RA Name:
13. RA Phone:

**Section A – 2: Facility Information**	**A – 2**
1. Facility Type (1-6): 1 = Teaching, 2 = Regional, 3 = Metropolitan, 4 = Municipal, 5 = District, 6 = Hospital	1. ______
2. Facility Ownership (1-5): 1 = Government, 2 = Quasi government, 3 = Private, 4 = CHAG, 5 = Other faith based, *indicate*	2._______
3. *Exclusive* Breast clinic [Y/N]:	3._______
4. Sees breast concerns = [Y/N]:	4._______
5. *Exclusive* Cervical clinic [Y/N]:	5._______
6. Sees cervix/gyn concerns [Y/N]:	6._______

Section B – 1: Number of Personnel

If none, indicate with “0”. *=MD

1. MD trained surgeon: ______
2. MD trained plast/reconst surgeon ______
3. MD trained general Ob/Gyn ______
4. MD Gyn/Onc: ______
5. MD trained Gen. Pract ______
6. Anesthesiologist: ______NurseAn _____S* ______C*
7. Radiologist: _____Tech ______S ______C
8. Pathologist: _____Tech ______S _______C
9. Oncologist: _____Nurse ______S* ______C*
10. Rad Onc: ______S ______C
11. Palliative Care: _______Nurse ______S* ______C*
12. Social worker: ______
13. General Nurse: (not incl above) ______
14. Public Health Officer: ______

**Section B – 2: Staffing for Services** Type (eg. Nurses/MD) + Number, 0=none Example: Nurses 5, MD gen pract 8)	**B – 2**
1. CBE	1. ___________
2. Breast Biopsy	2. ___________
3. Bimanual Pelvic Exam	3. ___________
4. VIA	4. ___________
5. VILI	5. ___________
6. Pap Smear	6. ___________
7. Colposcopy	7. ___________
8. Endocervical Curettage (ECC)	8. ___________
9. Cryotherapy	9. ___________
10. Thermoablation not with CinLuma	10. ___________
11. Thermoablation with CinLuma	11. ___________
12. LEEP	12. ___________
13. Cold Knife Cone	13. ___________
14. HPV Screening	14. ___________
15. HPV Subtype Screening (16/18)	15. ___________
16. HPV Vaccination	16.___________

**Section C – 1: Onsite Imaging and Labs** Answer Y1, Y0, or N.	**C – 1**
1. Breast US only	1. ___________
2. Breast + Axillary (lymph node) US	2. ___________
3. Abdominal US (evaluate liver, kidneys)	3. ___________
4. Transabdominal Pelvic US	4. ___________
5. Transvaginal Pelvic US	5. ___________
6. X-ray	6. ___________
7. Intravenous Pyelogram (IVP)	7. ___________
8. Mammography **complete C2 if yes**	8. ___________
9. Bone Scan	9. ___________
10. CT Scan	10. ___________
11. MRI	11. ___________
12. PET Scan	12. ___________
13. Complete Blood Count	13. ___________
14. Chemistry Panel	14. ___________
15. Liver Function Test	15. ___________

**Section C – 2: Mammogram Sub Survey**	**C – 2**
1. Keep records [Y1, Y0, or N]	1. ___________
2. Number mammos per month (1-4): (1 = 1-10,2 = 11-30,3 = 31-100,4 = >100)	2. ___________
3. Free to patient [Y1, Y0 or N]	3. ___________
4. If Y0 or N, what is the cost? (1=<100 2=100-500 3=>500)	4. ___________
	5. ___________
5. Insurance covered [Y1, Y0 or N]	6. ___________
6. Which (1=NHIS or 2=Private, list):	7. ___________
7. If “1”, 100% by NHIS? [Y1, Y0 or N]	
8. Who interprets? (1=Outside facility 2 = in house non rad 3 = in house cons rad 4 = in house spec rads 5 = other, list)	8. ___________
9. If answered “1”, where does the external review occur? (facility name/location & country)	9. ___________
10. Time for results (1-3) (1=<2 weeks,2=2 weeks-1 month,3=>1month)	10.__________

**Section D – 1: Pathology – Breast Cancer**	**D – 1**
Answer Y1, Y0, or N.	
1. Pathology avail for BC diagnosis?	1. ________
2. If Y, where services performed? 1=In-House, 2=external/Send-Out)	2.__________
3. If answered "2", where does the external review occur? (facility name/location & country)	3.__________
4. Result time (1=<2w, 2=2w-1m; 3=>1month)	4.__________
5. ER IHC	5.__________
6. PR IHC	6. __________
7. HER2/Neu IHC	7.__________
8. HER2/Neu FISH	8.__________
9. Special Stains Result time (1=<2w, 2=2w-1m; 3=>1month)	9.__________

**D – 2: Pathology – Cervical Cancer**	**D – 2**
Answer Y1, Y0, or N.	
1. Pathology avail for CC diagnosis?	1. __________
2. If Y, where services performed? 1=In-House, 2=external/Send-Out)	2.__________
3. If answered "2", where does the external review occur? (facility name/location & country)	3.__________
4. Result time (1=<2w, 2=2w-1m; 3=>1month)	4.__________

**E – 1 : Breast Cancer Diagnosis**	**E – 1**
Answer Y1, Y0, or N.	
1. CBE, for Screening	1. __________
2. CBE, for Workup	2.__________
3. Biopsy FNA	3.__________
4. Biopsy Core Needle	4.__________
5. Biopsy Excisional (surg)	5.__________
6. Biopsy Incisional (surg)	6.__________
7. Biopsy Skin Punch (inflamm bc)	7.__________
8. Biopsy Image-guided, Mammo	8.__________
9. Biopsy Image guided, US	9.__________
10. Genetic testing for patients who are high risk?	10._________

**E – 2: Breast Cancer Staging**	**E – 2**
1. Facility perform staging? [Y1, Y0 or N] *If yes, which modalities?*	1.__________
2. Clinical Exam [Y1, Y0 or N]	2.__________
3. Imaging [Y1, Y0 or N]	3.__________
4. If imaging is used, indicate which modalities (can list >1). (1=XR, 2=CT, 3=US, 4=MRI, 5=PET)	4.__________
5. Pathology [Y1, Y0 or N]	5. __________

**E – 3: Breast Cancer Surgery**	**E – 3**
1. Facility perform Breast surgery?	1. __________
(If no, proceed to #12.)	
2. WLE/Lump [Y1, Y0 or N]	2.__________
3. Mastectomy [Y1, Y0 or N]	3.__________
4. Level I/II axillary dissection [Y1, Y0 or N]	4.__________
5. SLN surgery? [Y1, Y0 or N]	5.__________
(If no, proceed to question #8)	6. __________
6. Blue Dye [Y1, Y0 or N]	
7. Radio isotope (Y1, Y0 or N; write which, if known)	7.__________
8. Facility offer Reconstruction?	8.__________
(If no, proceed to question #12)	
9. Expander/implant [Y1, Y0 or N]	9. __________
10. Rotation flap [Y1, Y0 or N]	10._________
11. Free flap[Y1, Y0 or N]	11._________
12. Facility offer ovarian ablation?	12._________
(If no, proceed to section E – 4.)	
13. Surgical oophorectomy [Y1, Y0 or N]	13._________
14. Ovarian irradiation [Y1, Y0 or N]	14._________
15. Medical Ablation with leuprolide	15._________
16. Medical Ablation with goserelin/zoladex	16._________

**E – 4: Breast Cancer Chemotherapy**	**E – 4**
Answer Y1, Y0, or N.	
1. Facility offer chemotherapy for BC treatment? *(If no, proceed to section E – 5)*	1. __________
2. Capecitabine	2.__________
3. Carboplatin	3.__________
4. Cisplatin	4.__________
5. Cyclophosphamide	5.__________
6. Docetaxel	6. __________
7. Doxorubicin/Adriamycin	7.__________
8. Epirubicin	8.__________
9. Gemcitabine	9. __________
10. Lapatinib	10._________
11. Methotrexate	11._________
12. Paclitaxel	12._________
13. Trastuzumab	13. _________
14. Vinorelbine	14. _________
15. 5-Fluorouracil	15. _________
16. Other, please specify	16._________
17. List Combinations used:	17._________

**E – 5: Endocrine Therapy for Breast Cancer**	**E – 5**
Answer Y1, Y0, or N.	
1. Facility offer endocrine therapy? *(If no, proceed to section E – 6)*	1. ________
2. Anastrozole	2. ________
3. Ethinyl estradiol	3. ________
4. Exemestane	4. ________
5. Fluoxymesterone	5. ________
6. Fulvestrant	6. ________
7. Goserelin	7. ________
8. Letrozole	8. ________
9. Megestrol acetate	9. ________
10. Tamoxifen	10. ________
11. Toremifene	11. ________
12. Other	12. ________

**E – 6: Onsite Radiation Therapy for BC**	**E – 6**
Answer Y1, Y0, or N.	
1. Facility offer radiation therapy? *(If no, proceed to section E – 7)*	1. ________
2. Whole Breast	2. ________
3. Chest Wall	3. ________
4. Regional Nodal	4. ________

**E – 7: Follow Up and Outreach for BC**	**E – 7**
Answer Y1, Y0, or N.	
1. Long term follow up? *(If no, proceed to #6.)*	1. ________
2. History and physical exam (note frequency)	2. ________
3. Annual mammogram	3. ________
4. Laboratory assessment (if so, what labs)	4. ________
5. Other (please explain)	5. ________
6. Does facility keep a BC Registry?	6. ________
7. Does facility conduct BC outreach? *(If no, proceed to #14.)*	7. ________
8. Education on risk factors	8. ________
9. Education on signs/symptoms	9. ________
10. Educating breast cancer treatable/cureable	10. ________
11. Teaching how to breast self-exam	11. ________
12. Screening programs with CBE	12. ________
13. Screening programs with mammogram	13. ________
14. Palliative care?	14. ________
If yes, detail services in space below:	

**F – 1 Onsite Diagnosis & Treatment of Cervical Cancer**	**F—1**
Answer Y1, Y0, or N.	
1. Bimanual exam	1. ________
2. Speculum exam	2. ________
3. VIA	3. ________
4. VILI	4. ________
5. Pap smear	5. ________
6. HPV testing, by provider	6. ________
7. HPV testing, self-swab	7. ________
8. HPV testing, subtyping for high risk subtypes 16&18	8. ________
9. Colposcopy	9. ________
10. Cervical Biopsy	10. ________
11. Endocervical Curettage (ECC)	11. ________
12. Cryotherapy	12. ________
13. Thermoablation not with CinLuma	13. ________
14. Thermoablation with CinLuma	14. ________
15. LEEP	15. ________
16. Cold knife cone	16. ________
17. Facility offers "screen & treat" approach for possible precancerous lesions? (Y/N) *If yes, answer #17, 18, 19 If no, proceed to #20*	17. ________
18. List screening test(s) used	18. ________
19. List treatment(s) used	19. ________
20. Time between screening and treatment? (1= same day, 2= next day, 3= same week, 4= >1 week)	20. ________
21. If no, is following true? "Screening is performed and we refer for treatment" (Y/N)	21. ________
22. HPV vaccine?	22. ________
23. HIV testing?	23. ________

**F– 2: Cervical Cancer Staging**	**F – 2**
Answer Y1, Y0, or N.	
1. Facility perform staging? *If yes, which modalities?*	1. ________
2. Clinical Exam	2. ________
3. Imaging	3. ________
4. If imaging is used, indicate which modalities (can list >1). (1=XR, 2=CT, 3=US, 4=MRI, 5=PET)	4. ________
5. Pathology	5. ________
Are the following exams used at this facility for staging CC?	
6. Rectal Exam	6. ________
7. Cystoscopy	7. ________
8. Protoscopy	8. ________
9. Exam Under Anesthesia (EUA)	9. ________

F – 3: Cervical Cancer Surgery

Answer Y1, Y0, or N.

1. Facility perform gynecologic surgery?
   If no, proceed to section F – 4
2. Facility perform surgery for CC?
   Which surgeries are offered?
3. Simple hysterectomy
4. Radical hysterectomy
5. Trachelectomy
6. Bilateral pelvic lymph node dissection
7. Para-aortic lymph node dissection

F – 3

1. ________
2. ________
3. ________
4. ________
5. ________
6. ________
7. ________

F – 4: Cervical Cancer Chemotherapy

Answer Y1, Y0, or N.

1. Facility offer chemotherapy for BC treatment?
   (If no, proceed to section F – 5)
2. Bevacizumab
3. Carboplatin
4. Cisplatin
5. Docetaxel
6. Gemcitabine
7. Ifsofamide
8. Irinotecan
9. Mitomycin
10. Paclitaxel
11. Pemetrexed
12. Topetecan
13. Vinorelbine
14. 5-Fluorouracil
15. Other, please specify
16. List Combinations used:

F—4

1. ________
2. ________
3. ________
4. ________
5. ________
6. ________
7. ________
8. ________
9. ________
10. ________
11. ________
12. ________
13. ________
14. ________
15. ________
16. ________

F – 5: Onsite Radiation for CC

Answer Y1, Y0, or N.

1. Facility offer radiation therapy?
   (If no, proceed to section F – 6)
2. External beam
3. Brachytherapy

F – 5

1. ________
2. ________
3. ________

F – 6: Follow Up & Outreach for CC

Answer Y1, Y0, or N.

1. Long term follow up?
   (If no, proceed to #6)
2. History and physical exam (note frequency)
3. Cervical/vaginal cytology (note frequency)
4. Laboratory assessment (if so, what labs)
5. Other (please explain)
6. Facility have CC Registry?
7. Facility conduct CC outreach?
   (If no, proceed to #15)
8. Education on risk factors
9. Education on signs/symptoms
10. Educating CC treatable/curable
11. Teaching about recommended screening and HPV vaccination
12. Screening programs with VIA or VILI
13. Screening programs with Pap Smear
14. Vaccination programs for HPV
15. Palliative care?

If yes, detail services:

F – 6

1. ________
2. ________
3. ________
4. ________
5. ________
6. ________
7. ________
8. ________
9. ________
10. ________
11. ________
12. ________
13. ________
14. ________
15. ________

G—1 Referral Practices for Breast Cancer

All [1,2, 3, OR 4] + referral location

1. A patient with clinical diagnosis of BC: 1= Biopsy&path review at facility
   2= Biopsy taken at facility, path review performed other facility
   3= Biopsy&path review at other facility
2. A patient with confirmed path diagnosis of BC:
   1= Surg&non-surg treatment (chemo/radx) performed at facility
   2= Surg mgmt taken at facility, non-surg mgmt at other facility
   3= Non-surg mgmt at facility, surg mgmt at other facility
   4= Patient is referred to other facility for all treatment

G – 1

1. ________
   Location: ________
2. ________
   Location: ________

G—2 Referral Practices Cervical Ca

1. A patient with clinical diagnosis of CC:
   1= Biopsy&path review at facility
   2= Biopsy taken at facility, path review performed other facility 3= Biopsy&path review at other facility
2. A patient with confirmed path diagnosis of CC:
   1= Surg&non-surg treatment (chemo/radx) performed at facility
   2= Surg mgmt taken at facility, non-surg mgmt at other facility 3= Non-surg mgmt at facility, surg mgmt at other facility
   4= Patient is referred to other

G – 2

1. ________
   Location: ________
2. ________
   Location: ________

Breast and Cervical PIPES Question Guide and Data Dictionary
For all Yes/No questions (except as indicated), the answer should be recorded as one of the following: Y/1= Usually available (>80% of the time)
Y/0= Sometimes available (<80% of the time) N= not available
For example, a mammogram that exists but is only working, or only offered half of the year should be indicated as Y/0. If there is no mammogram at all, it should be indicated as N.

Section A – 1: Basic information.
1. Region name 2. District Name 3. Date 4. Respondent Name 5. Respondent Phone 6. His/Her Title 7. Name of Facility 8. Facility Address 9. GPS coordinates 10. Facility Phone 11. Facility Email 12.Research Assistant Name
13. Research Assistant Phone.

Section A – 2 Facility Information
1. Facility Type, write number.
    - 1 = Teaching 2 = Regional 3 = Metropolitan 4 = Municipal 5 = District 6 = Hospital
2. Facility Ownership, write number.
    - 1 = Government 2 = Quasi government 3 = Private 4 = CHAG 5 = Other faith based, *indicate*
3. Breast clinic: Does your facility have an exclusive breast clinic? Y/N
4. Sees breast concerns: Does your facility see patients who have breast concerns/complaints even if there is not an exclusive clinic (for example, in a general clinic)? Y/N
5. Cervical clinic: Does your facility have an exclusive cervical clinic? Y/N
6. Sees cervical concerns: Does your facility see patients who have cervical concerns/complaints even if there is not an exclusive clinic (for example, in a general or gyn clinic)? Y/N

Section B – 1: Number of Personnel
For this section indicate the number of personnel as asked. For some specialties, note the number of nurses, technicians (T), specialists (S), and consultants (C). For some specialties, the specialists and consultants should be MD trained, as indicated by *. Abbreviations are detailed below:
- MD= medical doctor
- Plast/reconst surgeon= plastic reconstructive surgeon
- Ob/Gyn= obstetrician-gynecologist
- Gyn/Onc= gynecologic oncologist (an ob/gyn who specializes in gynecologic cancers)
- Gen Pract= general practitioner
- NusrseAn= Nurse Anesthetist
- Rad Onc= radiation oncologist

Section B – 2: Staffing for Services
This section inquires about the training level of personnel in a facility. For example, there may be 5 nurses employed by a hospital who have received training on how to perform VIA, but the hospital they are working at might not offer the procedure. For each question, first specify the type of personnel (nurse, midwife, MD, etc.) then specify the number. Example: 3 nurses, 4 midwifes, and 2 doctors are trained in CBE at the hospital, write: “Nurses 3, Midwife 4, MD 2.” If no one is trained in the procedure at the hospital just write “0.” For details on the listed procedures and abbreviations, please refer to sections E – 1 and F – 1.

Section C – 1: Onsite Imaging and Labs
Ask if they have the listed diagnostic modalities <u>within their facility</u> by indicating Y/1 (usually available), Y/0 (sometimes available), or N (never available). Example 1: MRI always or usually available = Y/1. Example 2: XRay sometimes available (<80% of the time) = Y/0 Example 3: No CT scan = N. Abbreviations and descriptions are detailed below. If the answer to question #8 is Y1 or Y0, please complete Section C – 2. If the answer to question #8 is N, you can skip Section C – 2.
- US= ultrasound or ultrasonography
- Axillary= medical term for armpit
- Abdominal US= ultrasound to evaluate the abdominal organs, typically the liver and kidneys

- Transabdominal pelvic US= evaluation of the pelvic organs, typically the ovaries and uterus, through the lower abdominal wall
- Transvaginal pelvic US= evaluation of the pelvic organs with a probe in the vagina
- Intravenous pyelogram (IVP)= an x-ray exam that uses contrast to evaluate the kidneys, ureters, and bladder
- Mammogram= x-ray exam to evaluate breast tissue
- Bone Scan= imaging test that can evaluate for spread of cancer to the bones
- CT= computerized tomography, also sometimes called a "CAT scan"
- MRI= magnetic resonance imaging
- PET= positron emission tomography
- Complete blood count= blood panel that should include hemoglobin/hematocrit, white blood cell count, and platelet count
- Chemistry panel= blood panel that provides information about renal function and electrolytes including (but not limited to) sodium (Na), potassium (K), creatinine (Cr), and blood urea nitrogen (BUN)
- Liver function test= blood panel that provides information about liver function including (but not limited to) AST/GOT and ALT/GPT, bilirubin, albumin, and PT/INR

Section C – 2 Mammogram Sub Survey

To be completed only if mammogram service is <u>onsite</u> (answer to Section C – 1 #8 is Y1 or Y0).

1. Does the hospital keep records of the mammograms they perform?
2. Number of mammograms per month that the facility performs:
   a. 1 = 1-10
   b. 2 = 11-30
   c. 3 = 31-100
   d. 4 = >100
3. Is the mammogram free for the patient? If answer is Y1, skip #4, if answer is Y0 or N please answer #4.
4. If Y0 or N, how much does it cost?
   a. 1 = <100 GHC
   b. 2 = 100-500 GHC
   c. 3 = >500 GHC
5. Does insurance cover part or all of the cost? If no, please skip #6 and #7, proceed to #8.

6. Which insurance? 1 = NHIS 2 = Private, write. If the answer is "1", please answer #7. If the answer is "2", please proceed to #8.
7. If answer to #6 is "1," is is 100% covered by NHIS (free to the patient)? Y1, Y0, or N
8. Who interprets mammograms? List all applicable
   a. 1=Outside facility
   b. 2=In house non radiologist (such as the surgeon)
   c. 3=In house consultant radiologist
   d. 4=In house specialist radiologist
   e. 5=Other, please list.

9. If interpretation is at an external facility list (answer "1" to #8) facility name and location (including country).
   a. Example: Korle Bu Teaching Hospital, Accra, Ghana
10. How long does it take for the patient to receive their results?
    a. 1= <2 weeks
    b. 2= 2 weeks-1 month
    c. 3= >1month

Section D – 1 Pathology – Breast Cancer

1. Are pathology services available (either in house or as referral) for diagnosis of breast cancer (BC)? Y1, Y0, or N
2. If pathology is available, is it performed in the facility, or sent to an outside/referral facility?
   a. 1 = in house
   b. 2 = external/send out/referral

3. If pathology is sent out to an external facility list facility name and location (including country).
    a. Example: Korle Bu Teaching Hospital, Accra, Ghana
4. How long does it take for the patient to receive their results?
    a. 1= <2 weeks
    b. 2= 2w – 1 month
    c. 3= >1month
5. Is estrogen receptor (ER) immunohistochemistry (IHC) staining performed on the pathology? Y1, Y0, or N
6. Is progesterone receptor (PR) IHC staining performed on the pathology? Y1, Y0, or N
7. Is HER2/neu IHC staining performed on the pathology? Y1, Y0, or N
8. Is HER2/neu fluorescence in situ hybridization (FISH) performed on the pathology? Y1, Y0, or N

9. How long does it take for the patient to receive results about the special stains (#5-8)?
    a. 1= <2 weeks
    b. 2= 2w – 1 month
    c. 3= >1month

Section D – 2 Pathology – Cervical Cancer

1. Are pathology services available (either in house or as referral) for diagnosis of cervical cancer (CC)? Y1, Y0, or N
2. If pathology is available, is it performed in the facility, or sent to an outside/referral facility?
    a. 1 = in house
    b. 2 = external/send out/referral
3. If pathology is sent out to an external facility list facility name and location (including country).
    a. Example: Korle Bu Teaching Hospital, Accra, Ghana
4. How long does it take for the patient to receive their results?
    a. 1= <2 weeks
    b. 2= 2w – 1 month
    c. 3= >1month

Section E – 1 Breast Cancer Diagnosis

Ask if they have the listed tests/procedures sometimes performed to diagnose breast cancer by indicating Y/1 (usually available), Y/0 (sometimes available), or N (never available). Abbreviations and descriptions are detailed below:

- CBE= clinical breast exam
- For screening= exam performed for women **without symptoms or concerns**
- For workup= exam performed for **women who have noticed a mass/lump or have other concerns**
- FNA= fine needle aspiration
- Biopsy core needle= using a larger needle to take a sample of tissue
- Excision and incisional biopsies are both surgical procedures. Excisional= the entire mass is removed at the time of the procedure. Incisional= a small sample is taken from the mass for testing.
- Skin punch= a sample of skin is taken, specifically used for a type of breast cancer called inflammatory BC
- Image guided biopsies are biopsies taken with the help of imaging, either with mammogram or ultrasound.
- Genetic testing is usually a saliva test that can find out if someone is high risk for breast cancer due to a change in their DNA. Common high risk changes are BRCA1 and BRCA2.

Section E – 2 Breast Cancer Staging

This section is to understand how a hospital “works up” a patient with suspected breast cancer. Scenarios are detailed below. It is important to know that a hospital might perform clinical exam, imaging, and pathology for staging and it is okay to mark Y for both of those options.

1. Facility performs staging? Y1, Y0, or N
    a. This is asking if a facility does anything to better understand what is going on with a patient before treating them. If the facility simply refers patients to another hospital for further care after noticing something abnormal on an exam, they do NOT perform staging.
2. Clinical exam (CE): The facility performs a breast and axillary exam before proceeding with treatment.
3. Imaging: The facility also performs some type of imaging (US, xray, mammo, etc.) before treatment.
4. If imaging is used, indicate which modalities (can list more than 1).
    a. 1=XR

b. 2=CT
c. 3=US
d. 4=MRI
e. 5=PET

5. Pathology: The facility takes a biopsy before treatment.

Section E – 3 Breast Cancer Surgery

Ask if the following surgeries are performed onsite to treat breast cancer by indicating Y/1 (usually available), Y/0 (sometimes available), or N (never available). Abbreviations and descriptions are detailed below.

1. If the answer to this first question is N, you can proceed to question #12.
2. WLE= wide local excision, lump= lumpectomy, this is also sometimes called "breast conserving surgery."
3. Mastectomy= removal of the whole breast
4. Level I/II axillary dissection= surgery to remove lymph nodes in the arm pit tissue
5. SLN surgery= sentinel lymph node biopsy/surgery, this is surgery on the axilla that uses special techniques to only take a few lymph nodes, rather than all of them
6. SLN surgery performed with blue dye
7. SLN surgery performed with radio isotope, please write in what radio isotope is used if known
8. Reconstructive surgery, this is also known as "plastic surgery." It is surgery done to reconstruct/recreate the breast after removal. If the answer to this first question is N, you can proceed to question #12.

9, 10, and 11 are different types of reconstructive surgery.

12. Ovarian ablation can be used as a treatment for "hormone positive" (ER/PR positive) breast cancer. Ovarian ablation can be accomplished with surgery, irradiation, or medication (questions 13-16).

Section E – 4 Breast Cancer Chemotherapy

If the answer to this first question is N, you can proceed to section E – 5. Questions 2 – 15 are all different names of chemotherapy medications. If the hospital offers another medication that is not listed, please write in on #16. For number 17, list what combinations (if any) the hospital uses (common examples are AC, AT, CMF, FAC, and CAF).

Section E – 5 Endocrine Therapy for Breast Cancer

Endocrine therapy is treatment for "hormone positive" (ER/PR positive) breast cancer. If the answer to this first question is N, you can proceed to section E – 6. Questions 2 – 11 are all different names of endocrine therapy medications. If the hospital offers another medication that is not listed, please write in on #12.

Section E – 6 Onsite Radiation Therapy for Breast Cancer

If the answer to this first question is N, you can proceed to section E – 7. Questions 2 – 4 are different types of radiation performed for breast cancer.

Section E – 7 Follow Up and Outreach for Breast Cancer

1. After a patient has been successfully treated for breast cancer, does the hospital continue to monitor them to watch for recurrence and help treat side effects from treatment? If no, proceed to #6.

2 – 5. You can answer yes to several of these questions. History and physical means the hospital sees the patient for a clinic visit to see how they are doing and perform an examination. Patients might also have mammograms, laboratory studies, or other tests performed for monitoring. If 'other' state which other modality.

6. A breast cancer registry is a record of patients diagnosed with breast cancer and treatments received.
7. Breast cancer outreach is education with the surrounding community about breast cancer, and might include screening programs/camps to help identify patients with concerns. If answer is no, proceed to #14.

8 – 13. Different types of outreach that a hospital might perform.

14. Palliative care is care to improve "quality of life" for patients with challenging to treat diseases, such as cancer. Palliative care might include things like assistance with pain control, physical therapy and rehabilitation after treatment, and counseling.

Section F – 1 Onsite Diagnosis and Treatment of Cervical Cancer

1. Bimanual exam= a healthcare provider places one hand inside the vagina to feel the cervix and one hand on the patient's lower abdomen to feel for masses or abnormalities of the cervix, uterus, and ovaries.
2. Speculum exam= exam that uses a speculum (metal or plastic duck-bill shaped instrument) to visualize the cervix.

3. VIA= visual inspection with acetic acid= an exam to look for precancerous or cancerous changes of the cervix
4. VILI= visual inspection with Lugol's iodine= an exam to look for precancerous or cancerous changes of the cervix
5. Pap smear= an exam to look for precancerous or cancerous changes of the cervix, this requires pathology 6 – 8. HPV= human papilloma virus, the most common cause of cervical cancer
9. Colposcopy= exam of the cervix that uses a magnifying tool to look for abnormalities
10. Cervical Biopsy= taking a piece of tissue from the cervix to look for or stage cancer
11. Endocervical Curettage (ECC)= taking scrapings of tissue from the cervix to look for cancer
12. Cryotherapy= treatment of precancerous cervical changes with very cold temperatures
13. Thermoablation not with CinLuma= treatment of precancerous cervical changes with very hot temperatures
14. Thermo-ablation with CinLuma= treatment of precancerous cervical changes with very hot temperatures
15. LEEP= loop electrosurgical excision procedure= surgical procedure for pre-cancerous or low grade CC
16. Cold knife cone= surgical procedure for pre-cancerous or low grade CC
17. "Screen and treat" is a way to manage pre-cancerous changes to the cervix. If a woman has an abnormal screening test, the recommendation is to treat as soon as possible.
    - If the answer is yes, answer # 18, 19, and 20 and skip #21
    - If no then proceed to #21.
18. Common screening tests are pap smear, VIA, and VILI
19. Common treatments are cryotherapy, thermoablation, and LEEP
20. How long between the abnormal screening test and the treatment? 1= same day, 2= next day, 3= same week, 4= >1 week
21. If answer to #17 was no, do they perform screening, then refer to another facility if an abnormality if found?
22. Does the facility offer the HPV vaccine?
23. Does the facility offer HIV testing?
24.

Section F – 2 Cervical Cancer Staging

This section is to understand how a hospital "works up" a patient with suspected cervical cancer. Scenarios are detailed below. It is important to know that a hospital might perform clinical exam, imaging, and pathology for staging and it is okay to mark Y for all of those options.

1. Facility performs staging? Y1, Y0, or N
    a. This is asking if a facility does anything to better understand what is going on with a patient before treating them. If the facility simply refers patients to another hospital for further care after noticing something abnormal on an exam, they do NOT perform staging.
2. Clinical exam (CE): The facility only performs a speculum and pelvic exam before proceeding with treatment.
3. Imaging: The facility also performs some type of imaging (US, CT, etc.) before treatment.
4. If imaging is used, indicate which modalities (can list more than 1).
    a. 1=XR
    b. 2=CT
    c. 3=US
    d. 4=MRI
    e. 5=PET

5. Pathology: The facility takes a biopsy before treatment.

There are some additional examinations that may be performed to stage cervical cancer. Ask the hospital if they perform these exams/procedures specifically for cervical cancer.

6. Rectal exam to ensure tumor has not invaded the rectum.
7. Cystoscopy= evaluation of the bladder to see if there is tumor invasion
8. Proctoscopy= visualization of the rectum to evaluate for tumor
9. Exam under anesthesia= sedation to allow for patient comfort while thoroughly evaluating extent of tumor

Section F – 3 Cervical Cancer Surgery

Ask if the following surgeries are performed to treat cervical cancer by indicating Y/1 (usually available), Y/0 (sometimes available), or N (never available). Descriptions of the surgeries are detailed below.

1. Does the hospital perform any type of gynecologic surgery? (does not have to be for cervical cancer)

2. Does the hospital perform surgery specifically for cervical cancer?
3. Simple hysterectomy= removal of uterus and cervix, but not surrounding structures
4. Radical hysterectomy= removal of the uterus, cervix, upper part of vagina, and surrounding tissue and ligaments
5. Trachelectomy= removal of the cervix and upper vagina with preservation of the uterine body (fertility preserving).
6. Bilateral pelvic lymph node dissection= removal of lymph nodes in the pelvis
7. Para-aortic lymph node dissection= removal of lymph nodes around the aorta

Section F – 4 Cervical Cancer Chemotherapy
If the answer to this first question is N, you can proceed to section F – 5. Questions 2 – 14 are all different names of chemotherapy medications. If the hospital offers another medication that is not listed, please write in on #15. For number 16, list what combinations (if any) the hospital uses.

Section F – 5 Onsite Radiation Therapy for Cervical Cancer
If the answer to this first question is N, you can proceed to section F – 6. Questions 2 and 3 are different types of radiation performed for cervical cancer.

Section F – 6 Follow Up and Outreach for Cervical Cancer
1. After a patient has been successfully treated for cervical cancer, does the hospital continue to monitor them to watch for recurrence and help treat side effects from treatment? If no, proceed to #6.

2 – 5. You can answer yes to several of these questions. For #2 and 3, please list frequency of the follow up, for example “every year” or “every other year.”

6. A cervical cancer registry is a record of patients diagnosed with cervical cancer and treatments received.
7. Cervical cancer outreach is education with the surrounding community about cervical cancer, and might include screening programs/camps to help identify patients with concerns. If answer is no, proceed to #14.

8 – 14. Different types of outreach that a hospital might perform.
15. Palliative care is care to improve “quality of life” for patients with challenging to treat diseases, such as cancer. Palliative care might include things like assistance with pain control, physical therapy and rehabilitation after treatment, and counseling.

Sections G – 1 and G – 2 Referral Practices
The purpose of these sections is to understand the referral practices for hospitals. For example, do they perform the procedures to diagnose a patient with breast or cervical cancer then refer then to another place for treatment? These answers should make sense based on their responses to the survey. You should only select ONE answer for each question, based on what the hospital MOST OFTEN does.

1. Please answer with one of the following options. If any referral takes place, please write the name of the hospital they refer to and where it is located.

1= Biopsies and pathologic review are performed at respondent’s facility
2= Biopsies taken at respondent’s facility, pathologic review performed at another facility
3= Patient is referred to another facility for both biopsies and pathologic review

2. Please answer with one of the following options. If any referral takes place, please write the name of the hospital they refer to and where it is located.

1= Surgical and non-surgical treatment (chemotherapy and radiotherapy) is performed at respondent’s facility
2= Surgical management is performed at respondent’s facility and patient is referred to another facility for non- surgical treatment
3= Non-surgical treatment is performed at respondent’s facility and patient is referred to another facility for surgical management
4= Patient is referred to another facility for all treatment
